# Caring for Dementia Caregivers: Psychosocial Factors Related to Engagement in Self-Care Activities

**DOI:** 10.3390/bs13100851

**Published:** 2023-10-18

**Authors:** Xinyao Lin, Jerad H. Moxley, Sara J. Czaja

**Affiliations:** Center on Aging and Behavioral Research, Division of Geriatrics and Palliative Medicine, Weill Cornell Medicine, New York, NY 10065, USA; jhm2006@med.cornell.edu (J.H.M.); sjc7004@med.cornell.edu (S.J.C.)

**Keywords:** caregiving, health and well-being, social support, caregiver involvement, demographic

## Abstract

Caregivers often prioritize the needs of the care recipient and neglect their own health needs. It is imperative to understand the factors related to their self-care practices and engagement in self-care activities. The present study examined the extent to which dementia caregivers engaged in self-care activities, how this varied depending on caregiver characteristics, and whether self-care engagement mediated the relationship between social support and caregiver outcomes. The study utilized baseline data from a diverse sample of dementia caregivers (N = 243) who participated in a randomized trial evaluating a psychosocial technology-based caregiver intervention. Results showed that the dementia caregivers engaged in low levels of self-care activities and that their engagement varied based on the caregivers’ background characteristics (age, gender, race/ethnicity, relationship to the care recipient, and employment status). Less caregiver involvement (e.g., less ADL/IADL help provided and more caregiver preparedness) and more social support predicted higher self-care activity engagement. Self-care activity engagement served as a mediator, such that more social support predicted more self-care activities, which, in turn, were associated with more positive perceptions of caregiving and less caregiver burden and depression. The findings suggest a need for interventions that promote self-care engagement among dementia caregivers and underscore the importance of social support and caregiver preparedness to caregivers’ well-being.

## 1. Introduction

### 1.1. Dementia Caregivers and Health

According to the Alzheimer’s Association, about 6.7 million Americans are living with dementia, and this number is projected to more than double by 2060 with the growth of the aging population [1]. Individuals with dementia rely heavily on the care provided by family caregivers (e.g., spouse, adult child), with 30% of individuals with dementia receiving care from three or more family caregivers [2]. The current study refers to family caregivers (e.g., adult child, spouse) of individuals with dementia as “dementia caregivers”, who provide unpaid care.

Due to the high caregiving demands, dementia caregivers report having worse physical (e.g., chronic conditions) and psychological health compared to the caregivers of patients with other conditions. In fact, 74% of dementia caregivers report that they are concerned about their own health [1]. Dementia caregivers are at a higher risk for developing chronic health conditions (e.g., heart diseases, diabetes, stroke) compared to non-dementia caregivers and the non-caregiving population [3,4]. According to the Stress Process Model and other empirical research, dementia caregivers face stressors from heavy caregiving tasks and demands, a fact which is related to a high prevalence of anxiety, depression, cognitive decline, burden, caregiver-related stress and strain, and social isolation [5,6,7,8]. The Stress Process Model emphasizes the importance for interventions to address dementia caregivers’ stressors from caregiving and to promote self-care [7]. Unfortunately, caregivers often place the needs of the care recipient above their own, despite their own high prevalence of poor health. Thus, it is imperative to identify strategies that support dementia caregivers in prioritizing their own health and engaging in behaviors that promote their physical and psychological well-being. The present study examined the extent to which dementia caregivers engaged in self-care activities, how this varied based on the caregivers’ characteristics, and whether self-care engagement mediated the relationship between social support and caregiver outcomes.

### 1.2. Self-Care

Engaging in self-care activities enables dementia caregivers to maintain their own health while effectively caring for their loved ones. Self-care is conceptualized by Orem’s self-care theory as activities that individuals engage in to improve their physical and psychological health and well-being [9]. The Theory of Self-Management Behavior complements this idea by emphasizing the multifaceted nature of individuals’ actions to manage health conditions effectively [10]. Past studies have identified caregivers’ self-care needs to include health-promoting behaviors (e.g., sleep), leisure activities for stress management (e.g., reading), and resources for their own physical care (e.g., medication management, regular doctor visits) [11,12,13]. Although these studies investigated the components of self-care, they did not assess the actual level of engagement in self-care activities among dementia caregivers. The current study builds on the Caregiver Health Model, which emphasizes caregiver-health-promoting behavior as one of the crucial determinants of caregivers’ health [14]. To better understand dementia caregivers’ self-care profile and practices, the current study examined the extent to which dementia caregivers engage in self-care activities and the extent to which factors such as a caregiver’s background characteristics, level of involvement in caregiving, and social support influence their engagement in self-care.

### 1.3. Caregiving Involvement and Self-Care

Dementia caregivers provide more intense care compared to caregivers of older adults with other conditions [8]. Dementia caregiving involvement includes the following: assisting with daily living activities (ADL: e.g., bathing, feeding) or with instrumental activities of daily living (IADL: e.g., transportation, chores, managing financial affairs); managing behavioral symptoms (e.g., agitation, nighttime disturbance) or other health conditions and comorbidities of the care recipients (e.g., diabetes, cancer); arranging formal services (e.g., paid in-home care); and providing overall management and emotional support. Moreover, caregiving demands are time-intensive, with dramatic increases in caregiving hours as dementia progresses [15].

Studies have shown that caregiver involvement (e.g., time spent caregiving, use of formal services) varies based on age, gender, race/ethnicity, caregiver relationship, and employment status. Dementia caregivers are more likely to be older adults, over 65 years or older, women, and the adult, child, or spouse of the individual with dementia who report spending more caregiving time compared to their counterparts [1,16]. People who are not working are more likely to use formal support services [17]. Compared to White, Non-Hispanic caregivers, African American, Hispanic, and Asian American dementia caregivers report providing more care in terms of the number of hours per week, using less formal help and services, and having more caregiving demands. Caregiving involvement may affect a caregiver’s ability to engage in self-care activities [18]. The current study expands the literature by investigating whether self-care activity varies according to a caregiver’s background characteristics.

The level of involvement in caregiving can also cause the caregiver to prioritize their loved one’s needs over their own, which results in worsened health over time for the caregiver and increased risks of chronic disease, morbidity, and mortality [8,19,20]. Despite the potentially adverse relationship between caregiving involvement and caregivers’ health, few studies have investigated whether caregiver involvement (e.g., ADL/IADL help and formal use of care and services) is related to self-care activity engagement [18].

### 1.4. Social Support, Self-Care, and Caregiving Outcome

Available social support has been shown to promote health and well-being in dementia caregivers [21,22,23,24]. Further, both observational and intervention studies have shown that social support (e.g., social interactions, received support, satisfaction with support) from family and friends is positively related to caregivers’ engagement in self-care behaviors such as physical activity and sleep [21,22]. In addition, social intervention studies aimed at increasing health-promoting behaviors have shown improvements in caregiving outcomes such as burden [23,24]. However, to date, these interventions often focus solely on health behaviors rather than the holistic concept of self-care that consists of both physical and behavioral activities. Moreover, studies have not tested self-care as a mechanism in the relationship between social support and caregiving outcomes, despite evident associations between these factors. The present study addresses these gaps by comprehensively assessing self-care activities and including activities such as physical health check-ups, restorative activities, and sleep. To our knowledge, the current study is also the first to examine self-care as a mediator in the relationship between social support and caregiving outcomes.

### 1.5. The Current Study

The present study examines the extent to which dementia family-caregivers engage in self-care activities (physical health check-ups, restorative activity, sleep) and whether their engagement varies according to a caregiver’s background characteristics (age, gender, race/ethnicity, relationship to the care recipient, and employment status). We also investigated whether the level of involvement in caregiving (help with ADLs/IADLs, formal use of care and services, caregiver preparedness, and care recipient’s physical and psychological symptoms) and social support (social interaction, received support, and satisfaction with support) are associated with the caregivers’ engagement in self-care activities and whether self-care activity serves as a mediator in the relationship between social support and caregiving outcome (caregiver burden, depression, and positive aspect of caregiving).

### 1.6. Hypotheses

We hypothesized that dementia caregivers’ engagement in self-care activities would vary based on their background characteristics. Less caregiver involvement (more ADL/IADL help, more formal use of care and services, more caregiver preparedness, and less care recipient’s physical and psychological symptoms) would predict more engagement in self-care activities. We further hypothesized that more social support would predict more engagement in self-care activities and that self-care activities would mediate the relationship between social support and caregiving outcomes.

## 2. Methods

### 2.1. Participants

The participants (N = 243) were dementia caregivers ranging in age from 20 to 95 years old who participated in the Caring for the Caregiver Network study, which was a technology-based psychosocial randomized controlled trial that focused on caregiving skills to promote caregiver preparedness and efficacy and caregivers’ ability to manage the demands of caregiving and reduce caregiver burden.

The participants were recruited in the Miami region through community centers, support groups, flyers posted in clinics, advertisements via radio and newspaper, and participant referrals. The inclusion criteria included caregivers who had been providing care to a loved one (family member or friend) with Alzheimer’s disease or related dementia for at least 15 h a week, over 6 months, had contact with the care recipient at least five times a week, lived with or close to the care recipient, spoke English or Spanish, and were 18 years or older. The exclusion criteria included having a cognitive impairment based on the adjusted scores for the Telephone Interview for Cognitive Status [25] or having reported less than two caregiving-related stressors.

### 2.2. Procedure

This study was approved by the University of Miami Miller School of Medicine’s Institutional Review Board. Informed consent was obtained from each participant before the study. The intervention was a 6-month technology-based multicomponent psychosocial intervention delivered in both Spanish and English. The intervention was aimed at providing education, skills training, and social support, as well as reducing stress in the dementia caregivers included in the study. The current study used the baseline data collected by trained research assistants in the participants’ homes, as the intervention may have affected the variables of interest at follow-up assessments. All the study’s measures were administered by trained research assistants who completed the ratings based on the caregivers’ responses.

### 2.3. Measures

#### 2.3.1. Background Characteristics

The background measures included age, gender (one = male, two = female), race/ethnicity (the participants self-identified as Hispanic, White, or Black), relationship to the care recipient (spouse or other relationships), and employment status (full-time, part-time, homemaker, retired, or unemployed).

#### 2.3.2. Self-Care Activity

Self-care activity was assessed across three domains: physical health check-ups, restorative activities, and sleep. A latent construct of self-care activity was created within these domains, where higher levels of these variables indicated more self-care activities.

##### Physical Health Check-Up

The participants were asked whether, in the previous 6 months or year, they had experienced any of the following: had the time to see doctor when they thought they should; had slowed down and caught enough rest when they were sick; had lost or gained weight without meaning to; had seen their primary care physician for a routine check; had missed any scheduled doctor’s appointment; had their eyesight/hearing/teeth/blood pressure checked; and had a flu shot. The responses with “yes” were coded as one and “no” as zero. These ten items were summed, with two items (“lost or gained weight without meaning to” and “missed any scheduled doctor’s appointment”) being reverse coded. The scores ranged from zero to ten, with higher scores indicating more physical health check-ups.

##### Restorative Activity

The participants were asked about ten items [26] for the following question: “Over the past month, how often have you been able to spend time doing the following”. These items included the following: sports, quiet time by themselves, attending club/church/fellowship, hobbies, going out for meals with friends and relatives, visiting family and friends, doing other fun things with people, taking vacations out of town, being in parks and other outdoor settings, and unwinding at the end of the day. Each item was rated on a one (never) to four (everyday) scale and all the items were summed, with scores ranging from ten to fifty. A higher score indicated more engagement with restorative activities.

##### Sleep

Sleep was measured using the following item: “During the past month, how would you rate your sleeping quality overall?”. The participants responded on a scale from one = “very bad” to four = “very good”, with a higher score indicating better sleep.

#### 2.3.3. Caregiver Involvement

##### ADL/IADL Support

The participants were asked whether they helped their loved one with using the telephone, shopping, food preparation, housekeeping, doing laundry, traveling by car, bus, etc., medication, handling finances, getting into or out of a bed, chair or wheelchair, eating a meal, bathing, dressing from the waist up and the waist down, toileting, and grooming [27]. The responses with “yes” were coded as one and “no” as zero. The items were summed with scores ranging from zero to sixteen. A higher score indicated that more help was provided for ADL/IADL activities.

##### Use of Formal Care and Services

The use of formal care and services [28] were measured by the total number of services the caregiver or care recipient had received in the previous month from an agency or from someone paid privately to provide help including the following: a homemaker; a home health aide; a visiting nurse; going to a center for low-cost meals or having cooked meals delivered; getting transportation to places outside the home; attending senior daycare or senior day health program support groups, or visits to a physician/psychiatrist; seeing a counselor, psychologist, or clergy for help with personal or family problems; visiting an emergency room; being a patient in a hospital overnight or being admitted as a patient to a hospital; and having the care recipient be a resident in a nursing home. The responses with “yes” were coded as one and “no” as zero. The items were summed, with possible scores ranging from zero to fourteen. A higher score indicated more use of formal care and services.

##### Preparedness

An eight-item scale [29] assessed the caregivers’ view on how well prepared they were to provide care in the following areas: taking care of a family member’s physical or emotional needs, finding and setting up services, dealing with the stress of caregiving, making caregiving activities satisfying, responding to and handling emergency situations, getting help and information from the healthcare system, and taking care of the family member overall. The participants responded with zero = “not at all prepared” to four = “very well prepared”. The items were averaged, with a higher score indicating more preparedness.

##### Care Recipient’s Physical Symptoms

The caregivers were asked to give their best estimate of how often the care recipients experienced each of the following symptoms over the previous 7 days from zero = “not at all” to three = “very often (every day)”: lack of energy/fatigue, lack of appetite, pain, dry mouth, shortness of breath, nausea, difficulty sleeping, constipation/diarrhea, and confusion/difficulty concentrating [30]. These nine items were summed, with possible scores ranging from zero to twenty-seven. A higher score indicated that the care recipient had more physical symptoms.

##### Care Recipient’s Psychological Symptoms

The caregivers were asked to indicate how often the care recipients experienced each one of the following feelings during the previous 7 days from zero = “not at all” to three = “very often (every day)”: afraid, confident, worried or anxious, irritable, depressed, cheerful, hopeless, sad/blue, burden to others, angry, lonely, embarrassed about self, guilty, abandoned, and rejected [30]. These fifteen items were summed, with two items (“confident” and “cheerful”) being reverse coded. The possible scores ranged from zero to forty-five, with higher scores indicating that the care recipient had more psychological symptoms.

#### 2.3.4. Social Support

Social support was assessed across three domains: received support, social interaction, and satisfaction with support. A latent construct of social support was created within these domains, where higher levels of these variables indicated more social support.

##### Received Support

Received support was assessed using a four-item scale, adapted from a modified version of the Inventory of Socially Supportive Behaviors [31,32]. The participants were asked to rate the frequency of receiving tangible, emotional, and instrumental support from zero =“never” to three = “very often”. The items were averaged, with higher scores indicating more received support.

##### Social Interaction

Social interaction was measured using the Lubben Social Network Index [33]. This consisted of three items that assessed the participants’ social network size (family, friends, and neighbors), with each response ranging from zero = “none” to five = “nine or more”. The items were averaged, with higher scores indicating more social interaction.

##### Satisfaction with Support

Satisfaction with support [32] was measured using three items that assessed the caregivers’ satisfaction with the emotional, tangible, and instrumental support received, and one item that asked about the participants’ overall satisfaction with the received support, with each response ranging from zero = “not at all satisfied” to three = “very satisfied”. The four items were averaged, with higher scores indicating more satisfaction with the support received.

#### 2.3.5. Caregiver Outcomes

The caregiver outcomes were assessed across three domains: caregiver burden, positive aspects of caregiving, and depression. A latent construct was created within these domains, where a higher score indicated more positive caregiver outcomes (more positive aspects of caregiving and less caregiver burden and depression).

##### Caregiver Burden

The twelve-item Zarit Burden Interview [34] was used to assess the caregivers’ feelings about caring for the care recipient (e.g., “do you feel that your health has suffered because of your involvement with the care recipient”). The response for each item ranged from zero = “never” to four = “nearly always” and all the items were summed.

##### Positive Aspects of Caregiving

The caregivers were asked about their positive feelings about their caregiving experiences with an eleven-item questionnaire [35], which included statements such as “providing help to the care recipient has made me feel appreciated, important”, etc. The response for each item ranged from zero = “disagree a lot” to four = “agree a lot”. The items were summed, with a higher score indicating more positive perceptions of caregiving.

##### Depression

The caregivers’ depression was assessed using the ten-item CES-D (Center for Epidemiological Studies—Depression) scale [36], which asked participants to report symptoms over the previous week, with items such as “had trouble keeping my mind on what I was doing”, “I felt restless”, etc. The response for each item ranged from zero = “rarely or none of the time” to four = “most or almost all of the time”, and the items were summed, with a higher score indicating more depression symptoms.

### 2.4. Analyses

Descriptive statistics (mean, standard deviation) were derived for all the variables and background characteristics. Independent *t*-tests and ANOVAs using IBM SPSS Statistics (Version 28) examined whether the caregivers’ engagement in self-care activities (physical health check-up, restorative activities, and sleep) varied with each background characteristic (age, gender, race/ethnicity, relationship to the care recipient, and employment status). For descriptive purposes, all the variables of interest were coded so that higher scores represented a higher presence of the variable traits (e.g., higher scores would indicate a higher positive perception of caregiving, depression, and caregiver burden).

Structural equation models in mplus 8 [37] were used to examine the association between a caregiver’s involvement in caregiving tasks and social support and a caregiver’s engagement in self-care activities, as well as to examine whether self-care activity served as a mediator in the relationship between social support and caregiver outcomes. Latent variables were used for social support (three indicators: received support, social interaction, and satisfaction of support), self-care activity (three indicators: physical health check-ups, restorative activities, and sleep), and caregiver outcomes (three indicators: caregiver burden, positive perceptions of caregiving, and depression) in all the structural equation models. The latent variable related to the caregiving outcome was coded such that higher levels of these variables would indicate a more positive caregiver outcome (more positive perceptions of caregiving and less caregiver burden and depression). The indirect effects of the mediation model were computed by the products of a × b, where a was the coefficient estimate of the association between social support and self-care activity and b was the coefficient estimate of the relationship between self-care activity and caregiving outcome. The model fit was examined with the χ^2^ test statistic, the comparative fit index (CFI; compares the proposed model to a null model), the root mean square error of approximation (RMSEA; adjusts for sample size), and the standardized root mean square residual (SRMR; compares observed and predicted correlations).

## 3. Results

The study sample consisted of 83.6% female and 16.4% male participants, of which 32.4% were White, 45.1% were Hispanic, and 22.5% were African American. Overall, 40.6% of the participants were caregiving for a spouse. As for the caregivers’ employment status, 37.7% of them were retired, 27% worked full-time, 11.5% worked part-time, 7% were homemakers, and 16.8% were unemployed. With respect to their education, 6.6% of the caregivers had less than a high school diploma, 12.3% had a high school diploma, 56.1% had some college experience, 20.9% had a college degree, and 4.1% had a graduate degree. All the people with dementia were living at home.

Table 1 shows the means and standard deviations of all the variables of interest and the background characteristics. Overall, the caregivers engaged in low levels of self-care activities relative to the maximum possible score (physical activity check-up: *M* = 6.09, *SD* = 2.24, possible score range: 0–10; restorative activity: *M* = 17.74, *SD* = 8.26, possible score range: 10–50; sleep: *M* = 2.51, *SD* = 0.86, possible score range: 1 (very bad)–4 (very good)).

The differences in caregiver engagement in self-care activities (physical health check-ups, restorative activities, and sleep) according to their background characteristics (age, gender, race/ethnicity, relationship to the care recipient, and employment status) are reported in Table 2. As shown, the caregivers over the age of 65 reported more physical health check-ups (*t*(242) = −6.71, *p* < 0.001), an engagement in more restorative activities (*t*(241) = −2.54, *p* = 0.01), and better sleep (*t*(241) = −2.24, *p* = 0.03) compared to the younger caregivers (age < 65 years). The female caregivers reported fewer physical health check-ups compared to the males (*t*(242) = −2.29, *p* = 0.02). The spousal caregivers reported more physical health check-ups (*t*(239) = −4.30, *p* < 0.001); they were also significantly older compared to the non-spousal caregivers (*r* = 0.64, *p* < 0.001). There were significant between-group differences for race/ethnicity with respect to a caregiver’s engagement in restorative activities (*F*(2, 239) = 4.19, *p* = 0.02), such that the Hispanic caregivers engaged in less restorative activities compared to the White participants (mean difference = −3.45, *p* = 0.005). The retired caregivers reported more physical health check-ups (*F*(4, 239) = 7.32, *p* < 0.001) compared to the non-retired caregivers, and had more sleep (*F*(4, 238) = 2.87, *p* = 0.02) compared to the unemployed caregivers (mean difference = 0.5, *p* = 0.002). An exploratory analysis was conducted to examine the relationship between the education level and a caregiver’s self-care activities. The results showed that there were significant between-group differences in one’s engagement in restorative activities (*F*(4, 238) = 3.12, *p* = 0.02) according to their education level, such that people with a college degree engaged in more restorative activities compared to people who did not complete high school (mean difference = −7.05, *p*_Bonf_ = 0.03). No significant effects were found between a caregiver’s education level and their physical health check-ups (*F*(4, 239) = 0.65, *p* = 0.63) or sleep (*F*(4, 238) = 1.13, *p* = 0.34).

### 3.1. Caregiver Involvement and Social Support Predicting Engagement in Self-Care Activities

**Figure 1:**
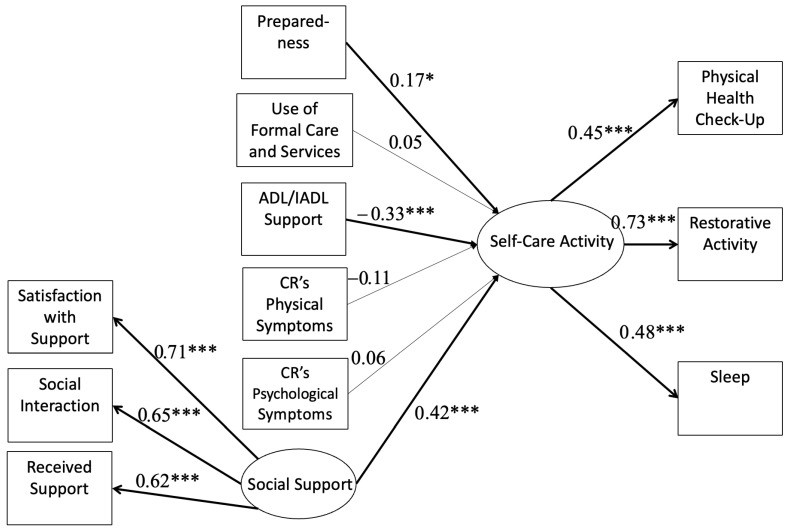
Structural equation model: caregiver involvement and social support predicting self-care activities. Note: Standardized estimates are reported. CR = care recipient. For simplicity, covariate (background characteristics) pathways are not depicted. Significant paths are bolded. * *p* < 0.05 and *** *p* < 0.001.

When examining the roles that caregiver involvement (help with ADLs/IADLs, formal use of care and service, caregiver preparedness, and care recipient’s physical and psychological symptoms) and social support played in the extent to which caregivers engaged in self-care activities, the structural equation model showed a good fit: χ^2^(28) = 45.51, *p* = 0.02, RMSEA = 0.05, 90% CI [0.02, 0.08], CFI = 0.99, and SRMR = 0.04. As predicted, more preparedness (*β* = 0.17, *p* = 0.03), less help with ADL/IADL (*β* = −0.33, *p*< 0.001), and more social support (*β* = 0.42, *p* < 0.001) were related to a greater engagement in self-care activities. Other caregiver involvement factors, such as the use of formal care and services (*β* = 0.05, *p* = 0.57), care recipient’s physical (*β* = −0.11, *p* = 0.20) and psychological (*β* = 0.06, *p* = 0.51) symptoms, were not related to engagement in self-care activities (Figure 1).

### 3.2. Self-Care Activity as A Mediator in the Relationship between Social Support and Caregiving Outcomes

**Figure 2:**
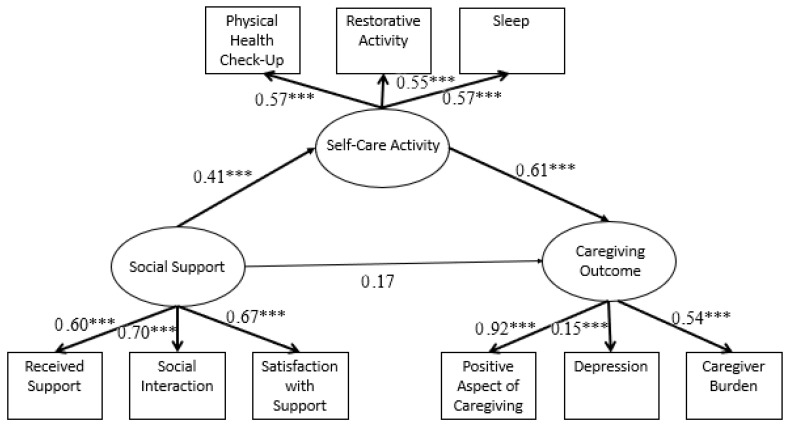
Structural equation mediation model: self-care activity as a mediator in the relationship between social support and caregiving outcomes. Note: standardized estimates are reported. Significant paths are bolded. *** *p* < 0.001.

The structural equation mediation model examining self-care activity as a mediator—χ^2^(23) = 66.86, *p* < 0.001, RMSEA = 0.09, 90% CI [0.06, 0.11], CFI = 0.90, and SRMR = 0.06—had a good fit. As expected, the results revealed that more social support was related to more engagement in self-care activities (*β* = 0.41, *p* < 0.001) and that more engagement in self-care activities was associated with better caregiver outcomes (*β* = 0.62, *p* < 0.001). There was no significant direct effect of social support on caregiver outcomes (*β* = 0.17, *p* = 0.08). However, as predicted, there was a significant indirect effect between social support and caregiver outcomes, mediated by self-care activity (*β* = 0.39, *p* = 0.03). In other words, more social support was related to more engagement in self-care activities, which, in turn, was associated with better caregiving outcomes (more positive perceptions of caregiving and less depression and caregiver burden) (Figure 2).

## 4. Discussion

Given dementia caregivers’ heavy caregiving demands, it is essential to understand the extent to which caregivers care for their own health and well-being. The goal of the present study was to examine the factors associated with engagement in self-care activities among dementia caregivers. The results showed that the caregivers’ engagement in these activities was relatively low; overall, the caregivers tended to neglect personal healthcare management tasks and had a low engagement in restorative activities. We also found that the engagement in self-care activities varied according to the caregivers’ background characteristics. A caregiver’s involvement (ADL/IADL help and caregiver preparedness) and social support also predicted caregivers’ self-care activity engagement. Moreover, self-care activity mediated the relationship between social support and caregiving outcomes.

While studies have examined the types of self-care activities that are needed by dementia caregivers and the barriers to engage in these activities [11,18], few have explored the extent to which caregivers engage in these self-care activities. As noted, our results indicate that dementia caregivers have a low engagement in self-care activities. These findings are important, as delineated in the Caregiver Health Model, because self-care is one of the critical determinants of caregivers’ health [14]. Given the importance of caregivers’ health with respect to both their own quality of life and also their ability to provide care, there is an urgent need for more interventions promoting the comprehensive engagement in self-care activities among dementia caregivers, as well as programs that prioritize caregivers’ self-care needs rather than solely focusing on addressing the needs of individuals with dementia. Moreover, healthcare providers should also consider assessing caregivers’ personal self-care routines. Further, it is important to recognize that self-care goes beyond just physical health activities, also encompassing restorative and supportive activities.

The current study also adds to the literature by examining whether self-care activity engagement differs based on caregivers’ background characteristics. Specifically, older, male, spousal, White, retired, and highly educated caregivers reported engaging in more self-care activities compared to their counterparts. We recognize that spousal and retired caregivers tend to be older and that older adults tend to utilize more healthcare services than younger adults. However, this finding highlights that certain caregiving groups are particularly vulnerable to a lack of engagement in these activities and require increased attention, targeted interventions, and support to promote self-care. When designing interventions, it is also crucial to tailor to caregivers’ own background characteristics (e.g., age, gender, race/ethnicity, education) rather than following the one-size-fits-all approach. It is also imperative for future self-care interventions to involve caregivers from diverse backgrounds in order to test their feasibility and efficacy. Furthermore, it is important to note that, while the older caregivers in this study tended to engage in higher levels of healthcare than the younger caregivers, their engagement in healthcare and restorative activities was still low.

Building on the Stress Process Model [7], in a manner consistent with the literature [8,15,19,20] and as hypothesized, caregiver involvement (help with ADL/IADL activities and caregiver preparedness) and social support were related to self-care activity engagement. The caregivers with higher levels of caregiver preparedness and more social support engaged in more self-care activities. Furthermore, those with lower levels of involvement in caregiving tasks also reported higher levels of self-care engagement. Dementia caregivers typically spend long hours each week providing care, such as helping the care recipients with ADL/IADL activities [15] and, thus, could have little to no time for self-care activities. On the other hand, caregivers that have been prepared for engaging in caregiving tasks and receive more support from family and friends could allocate more time to focus on their own well-being [38,39]. Dementia caregivers, especially those with high caregiving demands, should be encouraged to seek out additional assistance from others such as family or friends to help with daily caregiving tasks on days when they need to engage with their own healthcare activities or in order to free up time for some restorative activities. Policymakers and caregiving agencies should consider developing more programs to help dementia caregivers by providing support and training to increase caregiver preparedness. Self-care interventions can also include modules that help caregivers to connect with their existing social network members and facilitate peer social groups, as well as educational resources to enhance a caregiver’s preparedness to provide care.

Surprisingly, factors related to a caregiver’s involvement, such as their formal use of care and services and the care recipients’ physical and psychological symptoms, did not predict self-care activity. One possible explanation could be the overall low use of formal care and services (*M* = 1.43, *SD* = 1.18, possible scores = 0–14) and the low ratings of care recipient’s physical (*M* = 2.58, *SD* = 1.63, possible scores = 0–27) and psychological symptoms (*M* = 14.78, *SD* = 8.60, possible scores = 0–45). Moreover, the caregivers were asked about the care recipient’s symptoms in the previous week, whereas self-care activity questions asked for the caregivers’ engagement in restorative activities and sleep over the previous month. The lack of a significant relationship between these measures may be attributed to the inconsistency in the time-frame used for each measure and future studies should address such discrepancies.

To our knowledge, the current study is the first to identify self-care activity as a mediator in the relationship between social support and caregiver outcomes. The findings suggest that more social support was related to more self-care activity engagement, which, in turn, led to better caregiving outcomes (more positive aspects of caregiving and less depression and caregiver burden). In addition to examining engagement in self-care activities as an outcome, as previous studies have carried out [13,40], our findings suggest that self-care activity also serves as a mechanism for promoting caregivers’ positive well-being and reducing their burden. This finding also underscores the need for psychosocial interventions to focus on improving caregiver outcomes and promote self-care activity engagement. Agencies and services could offer a more person-centered approach with training, support, and advice on helping dementia caregivers connect with others and engage in self-care activities to promote positive caregiver outcomes.

A limitation is that the present study only used data collected at a one-time point which is correlational in nature and that causality cannot be confirmed. However, the findings can serve as a basis for future studies to examine the longitudinal relationships between caregivers’ involvement, social support, self-care activity, and caregiving outcomes to better determine the directionality of these relationships. Also, while the sample is relatively diverse in race and ethnicity, we did not collect information on other minority groups (e.g., Native Americans, Asian Americans). There are significant cultural differences between these race/ethnicity minority groups’ health and well-being [41], and future research should explore whether caregiver involvement, social support, self-care activity, and caregiving outcome vary according to race/ethnicity.

## 5. Conclusions

The present study contributes to the caregiving literature in the following ways: (1) by examining the extent to which dementia caregivers engaged in self-care activities and whether this varied according to the background characteristics of the caregiver; (2) by investigating the roles that caregiver involvement and social support play in predicting self-care activity engagement; (3) by assessing self-care activity comprehensively to include physical health check-ups, restorative activities, and sleep; and (4) by testing whether engagement in self-care activities acts as a mediator in the relationship between social support and caregiver outcomes, a contribution which, to our knowledge, was first accomplished by our study. The findings revealed that the dementia caregivers engaged in a low level of self-care activity and that their engagement varied based on the caregivers’ characteristics. Greater caregiver preparedness and social support and less help with ADL/IADL activities predicted more engagement in self-care activities. Furthermore, the engagement in self-care activities mediated the relationship between social support and caregiver outcomes. These findings call for more interventions to promote engagement in comprehensive self-care activities among dementia caregivers. Further to this, intervention studies that evaluate the feasibility of interventions in promoting self-care among caregivers should include caregivers from diverse backgrounds to help ensure a successful, broadscale implementation. This holistic examination is crucial for tailoring effective interventions that empower dementia caregivers to effectively balance their own health needs with their caregiving responsibilities.

## Figures and Tables

**Table 1:** Descriptives of the background characteristics and all the variables of interest.

Variables		Mean or %	Standard Deviation
Background Characteristics	Age	61.27	12.94
	Gender (% of Female)	83.60%	
	Race/Ethnicity		
	White	32.40%	
	African American	22.50%	
	Hispanic	45.10%	
	Relationship to the Care Recipient		
	Spouse	40.60%	
	Other Relationships	59.40%	
	Employment Status		
	Full-Time	27%	
	Part-Time	11.50%	
	Homemaker	7%	
	Retired	37.70%	
	Unemployed	16.80%	
Self-Care Activity	Physical Health Check-Up	6.09	2.24
	Restorative Activity	17.74	8.26
	Sleep	2.51	0.86
Caregiver Involvement	ADL/IADL Support	9.04	0.86
	Formal Use of Care and Services	1.43	1.18
	Preparedness	2.27	0.76
	Care Recipient’s Physical Symptoms	2.58	1.63
	Care Recipient’s Psychological Symptoms	14.78	8.60
Social Support	Received Support	6.25	2.74
	Satisfaction with Support	7.77	3.49
	Social Interaction	8.38	3.25
Caregiving Outcome	Caregiver Burden	19.09	8.29
	Positive Aspect of Caregiving	23.86	8.66
	Depression		

**Table 2:** Means, standard deviations, *t*-test, and ANOVA of self-care activity for each background characteristic.

	Physical Health Check-Up				Restorative Activity				Sleep			
	*M*	*SD*	*t* or *F*	*p*	*M*	*SD*	*t* or *F*	*p*	*M*	*SD*	*t* or *F*	*p*
Age												
Under 65	5.39	2.19	−6.7	<0.001	16.69	8.23	−2.54	0.01	2.41	0.90	−2.24	0.03
Over 65	7.21	1.83			19.43	8.08			2.67	0.78		
Gender												
Female	5.95	2.16	−2.3	0.02	17.67	8.36	−0.30	0.77	2.51	0.87	−0.12	0.91
Male	6.83	2.50			18.09	7.83			2.53	0.85		
Race/Ethnicity												
White	6.47	2.15	1.83	0.16	19.64	8.29	4.19	0.02	2.62	0.81	1.04	0.36
African American	5.80	2.06			18.26	7.85			2.53	0.86		
Hispanic	5.96	2.37			16.13	8.20			2.43	0.90		
Relationship to the Care Recipient												
Spouse	6.78	2.11	−4.30	<0.001	17.83	7.71	−0.25	0.80	2.63	0.79	−1.93	0.06
Other	5.56	2.19			17.56	8.66			2.42	0.90		
Employment Status												
Full-Time	5.83	1.92	7.32	<0.001	16.73	7.78	1.11	0.35	2.56	0.96	2.87	0.02
Part-Time	5.25	2.50			16.83	8.00			2.32	0.86		
Homemaker	5.29	2.17			18.74	8.29			2.59	0.71		
Retired	7.00	1.91			19.03	8.14			2.67	0.79		
Unemployed	5.37	2.59			16.70	9.36			2.17	0.83		

Note. *M* = means; *SD* = standard deviations; and *p* = *p*-values.

## Data Availability

The data presented in this study are available on request from the corresponding author. The data are not publicly available because the main outcome paper has not yet been published.
